# Atypical presentation of Sjogren’s syndrome with MALT lymphoma mimicking chronic venous ulcers

**DOI:** 10.1590/1677-5449.210003

**Published:** 2021-07-12

**Authors:** Marta Wasilewska

**Affiliations:** 1 Wroclaw Medical University – WMU, Department of Angiology, Hypertension and Diabetology, Wroclaw, Dolnoslaskie, Poland.

**Keywords:** leg ulcer, venous insufficiency, skin neoplasms, Sjogren’s syndrome, lymphoma, úlcera da perna, insuficiência venosa, neoplasias cutâneas, síndrome de Sjogren, linfoma

## Abstract

Chronic ulcerations of the lower extremities are quite a common condition amongst adults, most often caused by chronic venous insufficiency. Irrespective of the main underlying cause, chronic limb ulcerations are usually associated with significant symptoms, impairing daily functioning. Improper or delayed diagnosis and inadequate treatment increase the risk of serious complications, including limb amputations. Malignancies can develop secondary to chronic leg ulcers. About 2.4% of ulcers arising from chronic venous stasis undergo malignant transformation. Squamous cell carcinoma is the most common type of malignancy found in chronic leg ulceration biopsies. Basal cell carcinoma, sarcoma, and melanoma have all been documented infrequently. In the case described here, we found lymphoma of the marginal zone of mucosa-associated lymphoid tissue (MALT), which is an extremely rare cutaneous neoplasm of the lower extremities, but one that may have an association with autoimmune diseases.

## Resumo

Úlceras crônicas dos membros inferiores são uma condição bastante comum entre adultos, na maioria das vezes causadas por insuficiência venosa crônica. Independente da sua causa principal, úlceras crônicas dos membros estão geralmente associadas a sintomas significativos, prejudicando o funcionamento diário. O diagnóstico inadequado ou tardio e o tratamento inadequado aumentam o risco de complicações graves, inclusive o risco de amputação do membro. Malignidades podem se desenvolver secundariamente em úlceras crônicas da perna. Em torno de 2,4% das úlceras decorrentes de estase venosa crônica sofrem transformação maligna. O carcinoma de células escomosas é o tipo de malignidade mais comum que pode ser encontrada em biópsias de úlceras crônicas da perna. Carcinoma basocelular, sarcoma e melanoma foram raramente documentados. No caso aqui descrito, encontramos linfoma da zona marginal do tecido linfoide associado à mucosa (*mucosa-associated lymphoid tissue*, MALT), o qual é uma neoplasia cutânea dos membros inferiores extremamente rara mas que pode estar associada a doenças autoimunes.

## INTRODUCTION

Chronic ulcerations of the lower extremities are quite a common condition amongst adults. They are most often caused by chronic venous insufficiency. Other fairly common causes include atherosclerotic peripheral artery disease, peripheral neuropathy, and diabetes mellitus. Uncommon causes include vasculitis, autoimmune diseases, Buerger's disease, lymphedema, hematological diseases, clotting disorders (e.g. antiphospholipid syndrome), infectious diseases, metabolic disorders, physical and chemical damage, drug reactions, and skin malignancies.^1^^,^^2^ About 2.4% of ulcers arising from chronic venous stasis undergo malignant transformation. Squamous cell carcinoma is the most common type of malignancy found in chronic leg ulceration biopsies. Other types of malignancies that can develop secondary to chronic leg ulcer are rare.^1^^-^^4^ In the case described here, we found lymphoma of the marginal zone of mucosa-associated lymphoid tissue (MALT) that had an association with coexisting Sjogren’s syndrome.

## CASE DESCRIPTION

A 66-year-old woman was admitted to the clinic in May 2020 due to bilateral chronic painful ulcerations of the lower extremities with onset 9 months previously. She also complained of eczema in the forearms and thighs. There were no accompanying fever or chills. In ambulatory care she was seen by a surgeon, an angiologist, and a dermatologist who established a diagnosis of venous ulcerations of the lower limbs. She was treated with antibiotics and local wound dressings for many months without effect.

The patient's medical history included long-term nicotinism, systemic arterial hypertension, chronic venous insufficiency of the lower limbs, and surgical resection of varicose veins of the right lower extremity. She had also undergone a left orbital tumor resection 3 years earlier. The histopathological examination conducted at the time had revealed MALT lymphoma.

**Figure 1:**
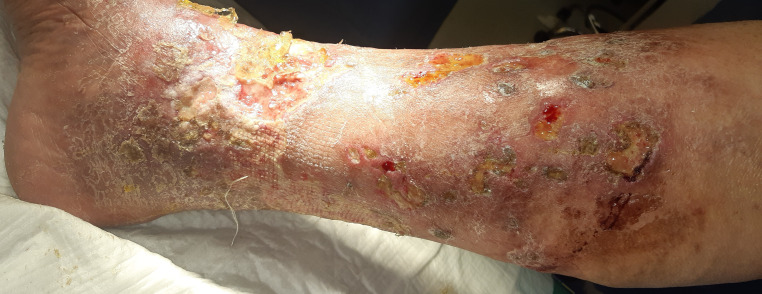
Numerous irregular infected ulcers of the right lower extremity.

**Figure 2:**
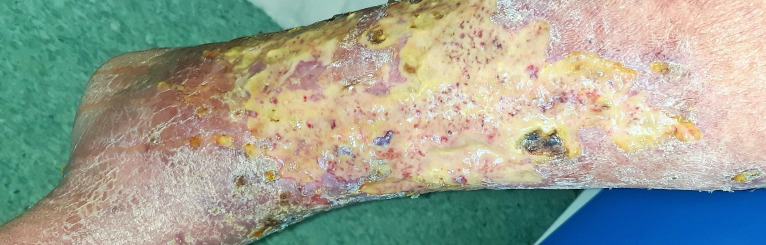
Extensive ulcers partially covered with necrotic tissue in the left lower extremity.

**Figure 3:**
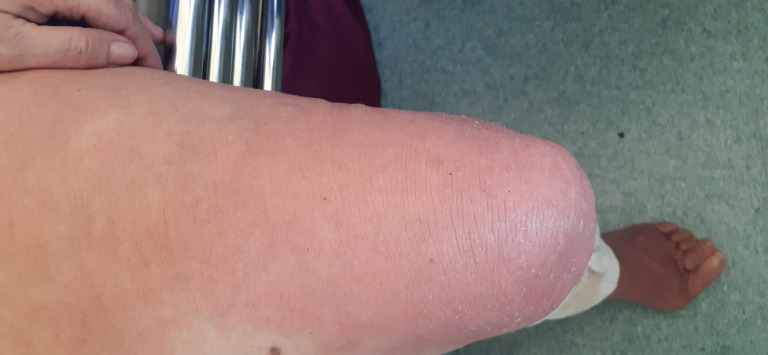
Eczema of the left thigh.

Physical examination of the lower limbs revealed extensive bilateral plastic edema, hyperpigmentation of the skin, and numerous large painful and irregular ulcers covered with fibrin and partially necrotic tissue (Figure 1 and 2). The skin of forearms and thighs was reddish, flaky, and itchy, with the appearance of venous eczema (Figure 3).

Laboratory tests revealed mild anemia, increased inflammatory parameters (CRP 88.7 mg/l), and deficiencies of folic acid and vitamins B12 and D3. Nonspecific abnormalities suggestive of autoimmune disorders were found: increased RF factors (150.7 IU/ml), decreased complement component C4 (<0.08 g/L), positive antinuclear antibodies (ANA) with positive anti-RO 52 antibodies in the profile, and a positive test for presence of cryoglobulins. Antiphospholipid antibodies, ANCA, and anti-CCP antibodies were negative. Viral infections such as HCV, HBV, HIV, CMV, and EBV were also ruled out.

Doppler ultrasound of the arteries of the lower extremities revealed normal high-resistance flows and ultrasound of the veins ruled out thrombosis. Abdominal ultrasound and chest X-ray were normal.

Due to nonspecific deviations in laboratory tests and the oncological history, autoimmune disease, vasculitis, and a neoplastic process were considered in the differential diagnosis. For this reason, a dermal-muscle section was taken from the largest leg ulcer. The labial gland was also collected for histopathological examination for Sjogren's syndrome. A PET scan examination was performed, ruling out recurrence of the lymphoproliferative process. Nevertheless, there were highlight “hot spots” in the lower limbs, which were interpreted as inflammatory lesions.

Histopathological examination of the leg ulcers showed lymphoplasmacytic infiltration with a CD20, CD43 immunohistochemical profile and restriction of Kappa K<L chains. These are morphological features of MALT lymphoma. The full quantitative and qualitative criteria of Sjogren's syndrome were found in the histopathological section from the labial gland. The exam revealed a very intense and advanced process with a high degree of inflammatory destruction of the parenchyma.

Due to the above results, the patient is currently under the care of an oncologist and rheumatologist.

## DISCUSSION

Chronic ulceration of the lower extremities affects about 1% of the adult population, 3.6% of people older than 65 years, and 5% of those aged over 80.^1^^,^^2^ Venous ulcers account for approximately 70% of cases of leg ulcers. They usually affect women and especially older people. Other risk factors are obesity, previous leg injuries, deep venous thrombosis, phlebitis, multiple pregnancies, congenital vein abnormalities, and calf muscle pump failure. Venous ulcers are usually located above the medial ankle, where venous pressure is highest. They have a tendency to recur and may persist for weeks to many years. They are associated with accompanying swelling and characteristic skin changes. Venous ulcers often produce a large amount of serous discharge. The skin around the ulcer is thickened, crusty, and hyperkeratotic with hyperpigmentation caused by hemosiderin staining. Occasionally, venous eczema develops, covering the area of the ulcer or the entire circumference of the lower leg.^1^ Most of the above symptoms were present in the patient described, which resulted in delayed diagnosis of other comorbid diseases.

Many neoplastic tumors types can present with skin ulceration as the first symptom. The most frequent are basal and squamous cell carcinomas. Other ulcerating tumors of the skin include malignant melanoma, metastasis, pseudoepitheliomatous hyperplasia, epithelioma, lymphomas, and sarcomas.^2^ Malignancies (especially squamous cell carcinoma) can also develop secondarily in chronic leg ulcers. This is probably caused by the continuously increased cell division in and around the ulcer.^2^ Venous stasis might also be a significant inducer of epidermal hyperplasia. The risk factors for development of malignancies of chronic ulcers are chronic inflammation, pre-existing osteomyelitis, decreased immune defense, susceptibility to malignancy, external factors (ultraviolet radiation), traumatic injuries, and chronic irritation.^3^^,^^4^ Malignant transformation of a long-lasting leg ulceration into a basal cell carcinoma has also been described, although it is rare. Few cases of sarcoma, lymphoma, or melanoma have been documented. However, in these cases, tumors were the cause of the ulceration being misdiagnosed as a chronic ulcer, rather than a complication of a chronic ulceration.^4^ Skin cancers may also arise de novo and mimic chronic venous ulcers in appearance. In such cases, the most frequent cutaneous malignancies are basal cell carcinoma, squamous cell carcinoma, and melanoma.^4^ Primary cutaneous lymphomas are approximately 65% T-cell in origin and 20–25% B-cells. Primary cutaneous B-cell lymphomas are classified into two major categories: indolent clinical course (which includes the marginal zone and follicle center) and intermediate clinical course (which includes intravascular large B-cell lymphoma and diffuse large B-cell lymphoma leg type).^5^

Hyperkeratotic granulation in the ulcer, alterations at the margin, unusual pain, abnormal excessive tissue granulation at wound edges, abnormal bleeding, or a protracted course despite appropriate treatment might be symptoms that indicate possible malignant transformation.^3^^,^^4^ This is why biopsy is recommended of nonhealing wounds after 6 weeks to 3 months.

The MALT lymphoma that was found in our patient is a low-grade lymphoma that develops from B cells, has greatest incidence between the ages of 50 and 60 years, and accounts for approximately 8% of all lymphomas. MALT lymphoma is characterized by a dense lymphoid infiltration that invades and usually destroys gastric glands. Lungs, intestines, lacrimal glands, tunica conjunctiva, thyroid gland, and skin are rarely affected.^6^^,^^7^ Primary cutaneous marginal zone B-cell lymphomas are characterized by the presence of numerous reddish papules, infiltrates or tumors, and are rarely ulcerated. These changes usually appear in the trunk and upper extremities and rarely in the lower extremities. They may be recurrent and sometimes spontaneous regression occurs. Involvement of the lymph nodes and internal organs is rarely described.^8^^,^^9^ In the patient described, no lymphatic cells in the lymph nodes, bone marrow or internal organs were identified, which indicates the primary nature of the cutaneous lymphoma.

Autoimmune diseases, especially Sjogren's syndrome, are risk factors predisposing to the development of MALT lymphoma.^7^ Occurrence of non-Hodgkin's lymphoma (NHL) is the most serious complication of Sjogren's syndrome. The risk of developing NHL in that group of patients was estimated to be 44 times greater than that observed in a comparable normal population. NHLs in Sjogren's syndrome occur preferentially in salivary glands and in other mucosa-associated lymphoid tissues, but also in lymph nodes and bone marrow.^10^

The patient had chronic venous insufficiency, which initially made diagnosis difficult and delayed it. Due to the unusual course of the disease and the ineffective treatment, a biopsy of the limb ulcer was performed, which should always be considered in such situations. In this patient, numerous non-specific autoimmune deviations were found. Moreover, the presence of cryoglobulins indicated the possibility of lymphoma. Therefore, in this case, Sjogren's syndrome was suspected, despite the fact that the patient did not present typical clinical symptoms of this disease. The decisive examination was the histopathological assessment of the salivary gland, which confirmed the autoimmune disease.

The permission of Ethical Committee in our institution is not required in this type of article (case report).

The patient’s informed consent was obtained for publication of this case report.
